# Patient preferences, knowledge and beliefs about kidney allocation: qualitative findings from the UK-wide ATTOM programme

**DOI:** 10.1136/bmjopen-2016-013896

**Published:** 2017-01-27

**Authors:** Andrea Gibbons, Marco Cinnirella, Janet Bayfield, Diana Wu, Heather Draper, Rachel J Johnson, Charles R V Tomson, John L R Forsythe, Wendy Metcalfe, Damian Fogarty, Paul Roderick, Rommel Ravanan, Gabriel C Oniscu, Christopher J E Watson, J Andrew Bradley, Clare Bradley

**Affiliations:** 1 Health Psychology Research Unit, Royal Holloway University of London, Egham, UK; 2 Department of Psychology, Royal Holloway University of London, Egham, UK; 3 Transplant Unit, Royal Infirmary of Edinburgh, Edinburgh, UK; 4 Division of Health Sciences, Warwick Medical School, Coventry, UK; 5 Statistics and Clinical Studies, NHS Blood and Transplant, Bristol, UK; 6 Department of Renal Medicine, Freeman Hospital, Newcastle upon Tyne, UK; 7 Organ Donation and Transplantation, NHS Blood and Transplant, Bristol, UK; 8 Regional Nephrology and Transplant Centre, Belfast Health and Social Care Trust, Belfast, UK; 9 Academic Unit of Primary Care and Population Sciences, Faculty of Medicine, University of Southampton, Southampton, UK; 10 Department of Renal Medicine, Southmead Hospital, Bristol, UK; 11 Department of Surgery, University of Cambridge, the NIHR Cambridge Biomedical Research Centre and the NIHR Blood and Transplant Research Unit in Organ Donation and Transplantation, University of Cambridge, Addenbrooke's Hospital, Cambridge, UK; 12 Health Psychology Research Ltd, Royal Holloway University of London, Egham, UK

**Keywords:** QUALITATIVE RESEARCH

## Abstract

**Objective:**

To explore how patients who are wait-listed for or who have received a kidney transplant understand the current UK kidney allocation system, and their views on ways to allocate kidneys in the future.

**Design:**

Qualitative study using semistructured interviews and thematic analysis based on a pragmatic approach.

**Participants:**

10 deceased-donor kidney transplant recipients, 10 live-donor kidney transplant recipients, 12 participants currently wait-listed for a kidney transplant and 4 participants whose kidney transplant failed.

**Setting:**

Semistructured telephone interviews conducted with participants in their own homes across the UK.

**Results:**

Three main themes were identified: uncertainty of knowledge of the allocation scheme; evaluation of the system and participant suggestions for future allocation schemes. Most participants identified human leucocyte anitgen matching as a factor in determining kidney allocation, but were often uncertain of the accuracy of their knowledge. In the absence of information that would allow a full assessment, the majority of participants consider that the current system is effective. A minority of participants were concerned about the perceived lack of transparency of the general decision-making processes within the scheme. Most participants felt that people who are younger and those better matched to the donor kidney should be prioritised for kidney allocation, but in contrast to the current scheme, less priority was considered appropriate for longer waiting patients. Some non-medical themes were also discussed, such as whether parents of dependent children should be prioritised for allocation, and whether patients with substance abuse problems be deprioritised.

**Conclusions:**

Our participants held differing views about the most important factors for kidney allocation, some of which were in contrast to the current scheme. Patient participation in reviewing future allocation policies will provide insight as to what is considered acceptable to patients and inform healthcare staff of the kinds of information patients would find most useful.

**Strengths and limitations of this study**

- Qualitative methods such as thematic analysis are well suited to understanding the beliefs underlying individual attitudes and opinions of the current kidney allocation system.
- The study interviewed a wide selection of participants, including those currently waiting for a transplant, participants who received a deceased donor or living-donor transplant and those whose transplant failed.
- The interviews were conducted with participants recruited to Access to Transplantation and Transplant Outcomes (ATTOM) so the results cannot be generalised necessarily to all renal patients or other organ allocation schemes.
- Only English-speaking participants were recruited so the results may not reflect fully the views of people of ethnic minority origin. Those patients deemed unsuitable for transplant listing were also not recruited.

## Introduction

Transplantation is widely viewed as the best treatment for most people with advanced chronic kidney disease (CKD).^1^ Although transplant rates are increasing, there continues to be a mismatch between supply and demand.^2^ ^3^ National kidney allocation policies aim to balance the competing goals of optimising outcomes and providing equity of access to donated organs, in a way that is acceptable to patients and healthcare professionals. In the current UK allocation scheme for kidneys from deceased heart-beating donors,^4^ ^5^ perfectly matched kidneys are prioritised for children (<18 years), patients with antibodies to numerous human leucocyte antigens (HLA) and patients homozygous for HLA-DR. Within these groups, longer waiting patients get priority. Imperfectly matched kidneys are offered to blood-group and HLA-compatible recipients using a points-based system taking into account waiting time and recipient age and HLA-mismatch, aiming to give younger patients better-matched kidneys. Other factors for which points are given include HLA-DR and HLA-B homozygosity, age difference between donor and recipient, blood-group match and geographical location of recipient and donor to minimise extracorporeal kidney storage time. When a donor is identified, kidneys are offered sequentially to wait-listed patients starting with the highest-ranked patient, then the next highest, until accepted. This scheme was introduced in 2006;^4^ ^5^ details are available online (http://www.odt.nhs.uk/pdf/kidney_allocation_policy.pdf). Since then, there have been significant changes in donor and recipient demographics, and other factors have emerged as predictors of post-transplant outcomes. In line with the objectives of the scheme,^4^ recent discussions suggest a shift in focus towards ‘transplant benefit’ and matching more carefully the donor and recipient. For this reason, the Kidney Advisory Group of National Health Service Blood and Transplant are reviewing the UK kidney allocation system.

Involving stakeholders in developing kidney allocation schemes helps ensure acceptability.^6^ Patients' perspectives and their preferences for factors determining allocation are important for assessing the acceptability of a system and managing patients' expectations of outcomes. Accounting for the outcomes most important to patients may improve patient satisfaction and their ability to make informed decisions regarding listing, but few studies have been conducted on the views of renal patients about kidney allocation schemes. Such studies have indicated broad agreement between factors considered important to patients and those used, such as HLA matching.^7^ ^8^ Most UK studies have used discrete choice experiments or questionnaire surveys,^8–10^ thus not allowing for further explanation of the reasoning and beliefs behind peoples' preferences or the opportunity to assess how patients interpret questions. Qualitative research, in contrast, provides a deeper understanding of the beliefs underlying individual attitudes and opinions and is capable of identifying unanticipated beliefs or preferences. The objectives of this study were to identify what patients know and think of the current UK kidney allocation system, and what factors they believe should influence allocation.

## Method

### Study context

This qualitative study was conducted as part of the Access to Transplantation and Transplant Outcomes (ATTOM) programme.^11^ ATTOM aimed to examine the reasons for disparities in transplant availability^12^ ^13^ and learn how to optimise UK transplant outcomes. Research nurses from all 72 UK renal units recruited participants from November 2011 to March 2013. The current study was conducted within a work-stream examining detailed patient-reported outcome measures in 651 patients fluent in English receiving differing treatments for stage 5 CKD, who completed questionnaires on quality of life and treatment satisfaction. Methods have been reported in detail elsewhere.^11^ The main aim of the interviews was to explore participant's questionnaire responses related to their quality of life and treatment satisfaction, and additionally explore participants' thoughts about how kidneys are allocated, which is the focus of this paper. Thematic analysis based on a pragmatic approach was used. This is a flexible approach not limited to any one epistemology that acknowledges there are differing ways of making sense of the world. Analyses were conducted in line with established guidelines.^14^

### Participants

To ensure inclusion of participants who reported differing levels of negative impact of their renal condition on their quality of life (QoL), participants were selected based on their Renal Dependent Quality of Life (RDQoL) questionnaire^15^ scores, completed 12 months following recruitment to ATTOM. The RDQoL is a 21-item disease-specific measure of the impact of CKD on QoL. The impact of CKD on various life domains, and the importance of these domains for QoL, are rated by participants. Impact is multiplied by importance to give a weighted-impact score for each domain. Average weighted-impact (AWI) scores are calculated by dividing the summed weighted-impact scores of each applicable domain by the number of applicable domains, to give a score between −9 (most negative impact) and +3 (most positive impact). Means and SDs were calculated from a subsample of 256 participants. Selecting participants with scores either above or below one SD of the mean allowed for consistent criteria to be used across treatment groups, and selected participants from a wide range of RDQoL AWI scores. Participants were not stratified by demographic variables, but the groups were representative of their cohorts for age, sex and ethnicity, although the living-donor kidney (LD) recipients interviewed were older than the average LD recipient.

Sixty participants were selected to take part (see online supplementary appendix 1); 40 agreed, and 38 were interviewed. Of the 20 who did not participate, 6 declined, 2 were too ill and 12 could not be contacted. Two agreed to participate but could not subsequently be contacted. Two interviews were excluded from analyses (1 wait-listed patient was removed from the list and 1 transplant recipient reported transplant failure at interview).

10.1136/bmjopen-2016-013896.supp1 supplementary appendices

### Interview schedule

An interview schedule was developed (box 1), guided by the published literature.^16^ Participants were asked about their knowledge of the kidney allocation system, and how they would like to see kidneys allocated in future. The interview also included a list of 13 factors that are or could potentially be used to determine allocation (table 1). The list included factors that are used in the UK scheme and those considered important by patients in previous research.^7–10^ ^16^ Current determinants of allocation were comprehensibly phrased; for example, cold ischaemia time was referred to as the ‘travelling distance between donor kidney and recipient’. Other factors were related to one another, such as likelihood of dying without a transplant and gain in life expectancy, but were assessed separately, in line with previous research.^7^ Participants were asked to rate the importance of these factors in deciding priority for who should be allocated a kidney from 0 (not at all important) to 10 (most important). All factors were rated by participants at the end of the interviews, to avoid influencing participants' opinions about the system. Participants were encouraged to elaborate on their answers and to think out loud when making their ratings.

Box 1 **Questions regarding kidney allocation from the wider interview schedule**

The broad questions were asked of all participants. The second set of questions include examples of questions used selectively to obtain further information or explanation from participants.

Broad questions

- Can you please tell me what you know of the current system that is used for allocating kidneys to people on the waiting list?
- Where did you learn about the allocation system?
- If you could decide how the allocation system works, how would you like to see the kidneys being allocated?
- Who do you think should decide who receives a kidney?
  Examples of prompts and probing questions
- What factors determine who gets a kidney?
- Can you tell me what you know of how tissue type and blood group influence kidney allocation? Can you tell me how the system tries to reduce the risk of rejection in other ways?
- Can you tell me what you know of how waiting time affects kidney allocation?
- What sort of person do you think is most likely to get a kidney? Why do you think that they have this advantage? Is this fair?
- Do you think that there are other people who should have an advantage? Who? Why?
- What sort of person do you think is least likely to get a kidney? Why do you think so? Is this fair?
- Do you think it is important that everyone has an equal chance of getting a kidney? Why?
- Do you think that sometimes people shouldn't have an equal chance? Who? Why?
- Where did you learn about the allocation system; healthcare staff? Other patients? The internet?
- Was the information you were given consistent with other information you received?
- What factors should be taken into account when deciding who receives a kidney?
- What factors do you think should be given priority? Can you explain why?

**Table 1:** Participants' ratings of how important they believe each factor to be in reaching a decision about who receives a kidney transplant as evidenced by mean importance scores

Ranking	Factor	Mean	SD	Range
1	HLA/tissue matching	8.66	1.76	1–10
2	Likelihood of dying without a transplant	8.30	1.72	5–10
3	Age <18 years of potential recipient	8.28	2.09	3–10
4	Blood-group match	8.09	2.33	1–10
5	Gain in quality of health	7.58	2.25	0–10
6	Travelling distance between donor kidney and recipient	7.51	2.76	0–10
7	Age 18–60 years	7.14	2.30	3–10
8	Gain in quality of life	6.73	3.12	0–10
9	Waiting time	6.59	2.31	0–10
10	Gain in life expectancy	6.48	2.97	0–10
11	Number of children of potential recipient	6.24	3.11	0–10
12	Other medical conditions	5.67	2.83	0–10
13	Age 60 years+	5.54	1.90	3–10

Factors rated from 0 (not at all important) to 10 (most important).

### Data collection

Semistructured telephone interviews were conducted between December 2013 and August 2014. All participants had been contacted previously by authors (AG or JB) when arranging completion of questionnaires. Postdoctoral research fellow (AG) conducted the interviews. She has qualitative research experience and formal training including the use of NVivo software (QSR International, US) for qualitative analysis. Participants were informed that the interview would explore their questionnaire responses related to their QoL and treatment satisfaction, to broaden the research team's understanding of participant experiences. The interview would also explore participants' thoughts about how kidneys are allocated (box 1). Participants agreeing to take part were phoned at an agreed time for interview.

### Data analysis

Interviews were audio-recorded and transcribed. Field notes were made by AG after every interview. Interviews averaged 52 min in length (range =28–91 min). Field notes were reviewed and transcripts read three times for familiarisation prior to analysis. Independent initial coding by AG of 5 interviews established major themes derived from the data which enabled development of a coding framework (AG, CB, MC). This showed significant levels of agreement on independent coding of the next five interviews (AG and JB). There was substantial coder agreement, and AG coded the remaining 26 interviews. The coding was completed in MSWord, then entered into NVivo10 software. Reiteration of earlier responses in later interviews indicated data saturation had been achieved.

## Results

### Participant characteristics

Table 2 shows participant characteristics. Participants were recruited through 11 UK transplant centres. The sample consisted of 10 deceased-donor (DD) kidney transplant recipients, 10 LD kidney transplant recipients, 4 participants whose transplant failed postrecruitment to ATTOM (Tx failed) and 12 participants wait-listed for a kidney transplant (WL). Four LD recipients received a transplant from a relative (1 parent donor, 3 adult-offspring donors), while 5 received an unrelated transplant through the national paired LD exchange scheme. The donors included in the scheme were relatives (n=1), spouses (n=3) or friends (n=1). One LD recipient received a transplant from a non-directed (altruistic) living donor. Two LD recipients were never wait-listed for a DD transplant. Wait-listed participants were waiting for an average of 41 months. More DD recipients reported having fewer educational qualifications, were from a wider range of transplant centres and had greater ethnic diversity than the other groups.

**Table 2:** Summary of demographic characteristics, time spent on dialysis and on waiting list for the four participant groups

	DD (N=10)	LD (N=10)	WL (N=12)	Tx failed (N=4)
Variable	M (SD)	M (SD)	M (SD)	M (SD)
Age (years)	52 (14.44)	53 (9.32)	53 (10.65)	53 (12.82)
Time on waiting list (months)	37 (34.48)	– (–)	41 (26.99)	13 (11.59)
Time on dialysis (months)	28 (24.59)	28 (31.67)	39 (31.53)	15 (0.82)
**Variable**	**N (%)**	**N (%)**	**N (%)**	**N (%)**
Sex (female)	5 (50%)	5 (50%)	5 (41.6%)	1 (25%)
Diabetes (yes)	3 (30%)	1 (10%)	1 (8.3%)	1 (25%)
Previous transplant failure (yes)	2 (20%)	4 (40%)	6 (50%)	1 (25%)
Treatment modality (pretransplant)				
Predialysis	3 (30%)	3 (30%)	2 (16.7%)	–
Peritoneal dialysis (PD)	1 (10%)	1 (10%)	2 (16.7%)	–
Haemodialysis (HD)	6 (60%)	6 (60%)	8 (66.6%)	4 (100%)
Marital status				
Single	2 (20%)	2 (20%)	3 (25%)	1 (25%)
Living with partner	1 (10%)	–	–	–
Married	5 (50%)	7 (70%)	7 (58.4%)	1 (25%)
Divorced/separated	1 (10%)	1 (10%)	1 (8.3%)	2 (50%)
Widowed	1 (10%)	–	1 (8.3%)	–
Education				
No qualifications	2 (20%)	1 (10%)	–	1 (25%)
Basic (O level/A level/NVQ 1–3)	4 (40%)	6 (60%)	9 (75.0%)	1 (25%)
Higher (degree/higher degree/NVQ 4–5)	4 (40%)	3 (30%)	3 (25.0%)	2 (50%)
Ethnicity				
White	7 (70%)	10 (100%)	8 (66.7%)	4 (100%)
Black	–	–	3 (25%)	–
Chinese	1 (10%)	–		–
Asian	1 (10%)	–	1 (8.3%)	–
Mixed	1 (10%)	–	–	–
Transplant centre				
Belfast	1 (10%)	2 (20%)	–	–
Birmingham	1 (10%)	–	2 (16.7%)	1 (25%)
Bristol	2 (20%)	–	1 (8.3%)	–
Cambridge	1 (10%)	1 (10%)	–	–
Cardiff	1 (10%)	–	1 (8.3%)	1 (25%)
Edinburgh	1 (10%)	4 (40%)	2 (16.7%)	–
Guys	–	–	–	2 (50%)
London West	1 (10%)	1 (10%)	3 (25%)	–
Newcastle	–	–	1 (8.3%)	–
Plymouth	2 (20%)	–	–	–
St. Georges	–	2 (20%)	2 (16.7%)	–

DD, deceased-donor-kidney-transplant group; LD, living-donor-kidney-transplant group; Tx failed, patients whose transplant failed; WL, patients wait-listed for a deceased-donor-kidney-transplant.

#### Main qualitative analyses

Three main themes emerged: certainty of knowledge; evaluation of the system and patient suggestions (see online supplementary appendix 2). See table 3 for themes and subthemes.

**Table 3:** Themes and illustrative quotations

Theme	Subtheme	Illustrative quotations
Certainty of knowledge of the allocation system	Perceived certainty	“Well I know that erm, the individual recipient has to match the donor with a blood type and antibody type and erm, I think there are 6 different numbers you've got to match with, or as near as a match with, before you can actually match up” (Man, WL pre-dialysis). “It goes by tissue matching. Basically like the lottery. I think you get, there's six things they got to match and the closest match, that's how they allocate the kidneys they give it to the closest match” (Man, DD transplant). “A lot of it goes on age and um compatibility so I believe the blood group is one of the first things” (Woman, (non-related) LD transplant). “I understand it's prioritises people who have been on the list for longer, waiting longer” (Man, WL CAPD). “I know they like to have as good an age match between donors and recipients as possible” (Woman, (non-related) LD transplant). “As far as I know obviously it's all computerised and it's the best match who gets them” (Woman, (non-related) LD transplant). “Well as I understand it um at (NHS) Blood and Transplant have a national allocation system. They, that essentially works on a combination of blood type and things like that and tissue typing, the kidneys are allocated on best match but that is flexed by need and time on the waiting list. So you have a combination of best match, overridden by someone who may have an urgent need or someone who spent an extremely long time on the waiting list” (Man, DD transplant).
	Knowledge uncertainty	“I don't know, I don't know what the system is” (Man, HD following failed DD transplant). *“*I don't, I haven't got a clue how they're allocated” (Man, (related) LD transplant). “I pretty much confess to a certain amount of ignorance because when I had my first one there was a points system and I was a young father, 40, and as I said to you before they wanted to try and transplant a few people early on, so I did, they claim, they say there's not a points system any more but I think probably some people's need is greater than others. And so I'm a little bit in the dark” (Man, WL HD)*.* “I don't know masses about it I've gotta be honest but my, my guess is they, erm they would look at how match(ed) the kidney is, they would look at how long people have been on the waiting list, they would look at probably age, I would say those are probably the key things, how long you've been on there, what kind of a match it's gonna be for you and what age, say how much kind of benefit you're gonna get from it” (Man, (related) LD transplant). “Well you get slightly different stories from slightly different people it has to be said. Um you know allegedly there's not a top of the list; right I mean allegedly it kind of works by a points system and all this kind of thing. But, um if you, I mean I only know in my personal circumstance I've been told that you know, I am now near the top of the list so there obviously is a priority sort of system” (Woman, WL predialysis).
Evaluation of the system	Perceived fairness	“I think the guidelines are very fair! I mean it's unfortunate if you've got a, a different metabolism as much as you're a different blood group or your whatever it is, the kidney doesn't match” (Man, DD transplant). “I think it's fair, you know I don't think anybody should be playing God and deciding ok this person is more needy therefore you get it. Sometimes it's unfair when you see like, somebody who is young, with family and stuff not getting one, but then I wouldn't want to be the person on that board deciding between that person and somebody else. The way they do allocate kidneys is much more neutral” (Woman, DD transplant). “Not knowing enough about how it's allocated, I would have to assume it's been set up in a good way that it is fairly fair … I would just have to assume that it's been set up in a good way so” (Man, (non-related) LD transplant). “(It's) probably not a fair system but I mean there's also things like I mean I know it shouldn't matter but I'm not sure geographically um, I mean allegedly it's all one system and you would get a kidney you know from the south of England or whatever but … um technically everybody should have an equal chance, I'm not entirely sure that's how it works. But I don't think that's necessarily wrong” (Woman, WL predialysis).
	Trust	“Well not knowing enough about how it's allocated, I would have to assume it's been set up in a good way that it is fairly fair but um, so yes I do not know all the ins and outs of the allocation system so I um, I would just have to assume that it's been set up in a good way” (Man, (non-related) LD transplant). “I suppose the consultant sees the patient and we should all trust the consultants that they're gonna be … if there's fraudulent or back-handed things going on then if we can see everything's kosher and they're making the right decisions for the right reasons” (Woman, predialysis DD transplant). “I hope that it is (a fair system). I don't have any knowledge of it but I hope that there is and they're not cheating me out of a kidney!” (Man, WL CAPD).
Patient suggestions	Medical priority	“I suppose priority would be um people who really needed it if they were ill. But then I think you have to look at, there's so many points to look at isn't there. You've gotta be healthy enough to receive it. It's no good going through a major operation if you … you've got to be fit enough to have it)” (Woman, HD following failed DD transplant). “But I think that because of the time and the money it costs I think the money should be best spent and the person most suitable to that kidney … the one that's most likely to be successful” (Man, (related) LD transplant). “And if it's so much to do with the individual then I guess it has to be um a match, a match basis like they're on cos they, ultimately whether it's going to work or not, medically, whether it's going to work or not, they don't just have to take the gamble medically on that and the best match has to be the thing that takes priority over everything. Always cos then the long-term prognosis is what they care about” (Woman, (non-related) LD transplant). “If you give a kidney to someone who doesn't best match it, it might only last them an hour or a day and then it's wasted. Someone who's a 100% match it could go forever and forever, you know it could last them forever” (Woman WL HD). “Well it shouldn't really matter because as I say it's only if it's a proper match. I mean obviously if you've got a guy with a couple of kids and a guy at 60, then it's the same match, a proper match then obviously the younger person should get it. But there's no point. They match the kidneys at present to the age group if they possibly can. I mean there's little point in putting a 60 year old kidney into a 20 year old is there? It's got to be a match” (Man, DD transplant). “I suppose like people with the worst kidney function obviously need a kidney first” (Man, DD transplant). “Well it's important to give it to the person who needs it most I think” (Man, DD transplant).
	Increasing life expectancy	“I mean if you had an 85-year-old who's been on the waiting list for goodness knows how long, and you've got a 30-year old and a kidney comes up and it's suitable for both of them, I would have thought that common sense would say the 30-year-old would get it” (Man, pre-dialysis, LD transplant). “You have to figure out how old are people who receive a kidney, they're more likely to have other health, health side effects than someone who's younger… and also if you're older you know, and you're like a widow or something then your social life is gonna be less than someone who's younger you know, who's just got married and has got kids and stuff like that” (Man, DD transplant). “I've lived my life. I'm 66 now and if there was a young person lying in bed beside me and two of us could, I would say give it to that, give it to that young life. Yes I would. But that's my personal thought. So I've lived my life, this guy's just starting out, you know” (Woman, DD transplant). “If you were something like 80 you wouldn't expect to live more than another ten years or something like that, where if you get it when you're 20, you could live another 60 years or something like that. So um, so yes I think it's quite important obviously if it can give a longer, if you're saying if it can give a longer lifespan then it's, um, that should be weighted into it” (Man, (non-related) LD transplant). “Well if that person was the main parent and they got two kids whatever, and if that person doesn't survive because they didn't receive a kidney, and then the kids have to go into care then you know there should be some priority over that [when] you've got two dependants, you know, little ones to look after (rather) than someone who hasn't got anyone (to look after)” (Man, DD transplant).
	Priority based on recipient factors	“I had a two year old child when I first had a transplant so I mean, you could argue that there was a child dependent on me but that, I‘m no more special than anyone else” (Woman, WL predialysis). “That's their fault (they had children)! If you choose to have children, you don't think well if I have more children I'm more likely to get a kidney transplant. No that's not fair” (Woman, pre-dialysis DD transplant). “I think everyone should be treated equally whatever age” (Woman, DD transplant). “I may have been more predisposed to say you know that younger ones should have priority, but since my father died I would have argued very strongly that he was entitled to just as many, you know as equal an opportunity as anyone else” (Woman, DD transplant). “I mean people might say oh yes you know she's a mum and she's got two young children, she should have priority whereas that's chap's 70 and shouldn't … I don't think that's a fair way to do it because who's to say, who's to judge that that person is more deserving of it, or would benefit from it more? I think that has to be a medical decision” (Woman, WL pre-dialysis). “Why should someone of a younger age be more entitled than someone of an older age?” (Man, (related) LD transplant). “If a young person has a chance of a decent life but then a middle aged man wants a chance of a decent life, he's got a family. He may be older, he's got a family and you know you can get to my stage of life and still have a family, still have a wife and you still want your shot! I think everybody should have an equal shot” (Man, WL HD).
** **	Deservingness	“You think about it people go on the transplant list, on the donor list then they should be worth more, get more marks than people who are not on it. So if you've been on the transplant list 20 years, and on the donor list for 20 years and you're an equal match as somebody that's not on the, on the donor list, you should give them priority” (Man, pre-dialysis DD transplant). “If people are grossly overweight, diabetic you know I mean a lot of, I mean you could argue the case all these people are sort of self-inflicted like you know?” (Man, LD transplant). “I think people uh, who are extremely ill and who deserve it, which for me would be young people, people with families, that kind of thing” (Man, WL pre-dialysis).

CAPD, continuous ambulatory peritoneal dialysis; DD, deceased-donor-kidney-transplant group; HD, haemodialysis; LD, living-donor-kidney-transplant group; Tx failed, patients whose transplant failed; WL, patients wait-listed for a deceased-donor-kidney-transplant.

#### Certainty of knowledge

Few participants reported detailed knowledge of the system, while many were unsure of how kidneys are allocated. Despite this, the majority correctly identified medical factors such as blood group and HLA typing as important, although all participants referred to HLA matching as ‘tissue matching’. Several participants correctly believed that recipient age was a factor, with younger people more likely to be prioritised. One DD recipient felt that there was an upper age limit for receiving a transplant, but was unsure of accuracy: “I think if you're over, I don't know, 60 or 70 you may be less likely to get one… I don't know whether I'm right in that or not, but that's my perception” (Woman, non-related LD transplant).

Some transplant recipients were correctly aware that waiting time is a factor, but very few were aware of how multiple factors combine to determine organ allocation. Some felt more knowledgeable about how factors inter-relate than others, but were still uncertain that their information was accurate. Participants whose transplants failed were knowledgeable about HLA typing and its importance in determining allocation. One participant in particular was very knowledgeable of the system, although he was uncertain about his accuracy: “I believe there's a points system and how that works specifically I don't really know. But I believe that the kidneys would be allocated on what would be the best match, medical match. I believe that children will probably take priority over older people. Whether that's true or not I don't know but that's what I understand. I believe if there is more than; if there's a really good match but more than uh, you know one person that it would be a match to, then that's when I believe the time you've been waiting on the waiting list would then come in. So if there's a donor kidney that would match four people it would go to the person who's been on the list the longest” (Man, on HD following failed DD transplant).

Some participants correctly believed that it is a national allocation system, with a small minority incorrectly believing that wait-listed participants move up the list in a sequential order. Three LD recipients were unsure of the system, but overall, LD recipients and WL participants were more familiar with the points system than the DD recipients who were beneficiaries of this system. WL participants were more likely to report seeking information, and participants whose transplants failed recalled being given information from surgeons and transplant coordinators, while those wait-listed for a transplant and LD recipients mentioned medical staff, seminars and information evenings more often. DD recipients reported receiving written information and researching the scheme on the internet. Most reported being satisfied with the information they received, although a fifth of participants expressed a wish for further information.

#### Evaluation of the system

When asked to describe the current system, 22 participants made evaluative judgements about its fairness. Patients regarded equity and fairness as synonymous. On the basis of their current knowledge of the system, 17 participants felt that the current system was fair, although 5 felt that it was not. They believed that some people were prioritised over others based on age and/or ethnicity, but did not necessarily believe that the system required change.

Several participants felt that they could trust their doctors, but also felt that if more information was available they could trust them more. One DD recipient believed very strongly that non-related living donation contradicts the information given about matching. Although details of the scheme are available online, this participant felt there was a lack of transparency: “… there's also this sneaking suspicion that someone somewhere gets to make a moral judgement on when the kidneys are handed out and to whom … I'd be much happier if I knew that it was absolutely numerical and somebody wasn't making a judgement call on it at some point … you've got no way of checking that because none of this is in the open” (Man, DD transplant).

#### Patient suggestions

When participants were asked how kidneys should be allocated, various factors were discussed, including matching, age, perceived medical need, dependants, waiting time and lifestyle factors. These factors were categorised under the themes medical priority, increasing life expectancy, priority based on recipient factors and deservingness.

#### Medical priority

When asked what factors should be important in deciding who receives a kidney transplant, the main factor recognised spontaneously to be of primary importance by 20 participants was matching (HLA and/or blood), within the context of medical priority. Participants felt so strongly about the importance of matching that many felt non-medical factors such as waiting time were irrelevant: “Your time on the transplant list is governed by if you're a perfect tissue match. That's it. You know you've got, … if it's not a perfect tissue match it's not going to take and if it's not going to take it's not worth having … you've just got to wait. You can't decide well I've waited 5 years I should have the next one” (Man, WL, on HD). Non-medical factors were considered secondary to having a successful, well-matched kidney: “I think what you're looking for is two things, you want the kidney to last and be as close a match as possible” (Woman, DD transplant). Nine participants also felt that people with the most perceived medical need or who are the most ill should be given priority: “Some people may be very, very ill and need the transplant … that should be taken into account” (Man, (related) LD transplant).

#### Increasing life expectancy

Participants held opposing beliefs about the importance of recipient factors in prioritising kidney allocation. These were often based on beliefs surrounding increasing life expectancy. Many participants felt that young people have yet to live their lives fully, and prioritising them allows them this chance: “I probably think that if you had to choose between a 20-year-old or a 90-year-old, I would probably give it to the younger one because they got their life to live whereas the 90-year-old has lived their life” (Man, DD transplant). At the same time, being older was considered to bring more risk of having other conditions that may complicate the success of a transplant and limit any gains in life expectancy.

#### Priority based on recipient factors

Although many participants felt that medical priority through matching was important, eight others felt it was unfair for anyone to receive priority based on recipient factors, including time on the waiting list, ethnicity, religion, younger age or having children: “I don't see why one person should have advantage over another” (Woman, (non-related) LD transplant). These participants felt that those who are younger should not receive priority, there should be an equal chance for all to receive a transplant: “everybody should get an equal chance, just because you're older, you still want to live, you still want to see your grandkids or your great-grandchild” (Woman, WL, on HD). Participants also noted that it would be unfair if those unable or unwilling to have children because of their renal condition were less likely to receive a transplant.

#### Deservingness

The issue of deservingness was also considered when discussing what factors should be used; two WL participants felt that patients who abuse their body via drugs or alcohol should be given less priority, while one DD transplant recipient believed that those willing to donate organs or who are already on the Organ Donor Register should be given priority if they require a transplant: “People who are willing to donate should be given a bit more priority than people who haven't, as an incentive (to donate)” (Man, DD transplant).

At the end of each interview, participants were asked to rate the importance of 13 factors in deciding priority for who should be allocated a kidney (table 1). Factors relating to medical priority were rated the highest; tissue matching was regarded as the most important, with the likelihood of dying soon without a transplant being considered the second most important factor. Being younger than 18 years of age was considered the third most important. No variable was considered unimportant, although having other medical conditions, being older than 60 years, and having children or dependants were rated as least important for kidney transplant allocation.

## Discussion

Although participants in our study were knowledgeable about some aspects of the current UK allocation system, they were not certain their knowledge was accurate. Participants may have been reluctant to place confidence in their knowledge, assuming that the interviewer had greater knowledge than them, though the woman interviewer made no claim to such knowledge. Interestingly, some groups such as those currently wait-listed were more knowledgeable than others. These participants had been wait-listed for 41 months on average and were more likely to report seeking information. In contrast, DD recipients may not have retained information about a system in which they are no longer involved. Although LD recipients received donations outside of the allocation scheme, they were more likely to be aware of the points system than DD recipients. Most were listed for a DD transplant before receiving a LD transplant and fewer LD recipients and WL patients reported having no qualifications. The DD recipients were from a wider range of transplant centres and had greater ethnic diversity. It may be that the differences in knowledge reflect these differences in sociodemographic factors, rather than treatment group, but further work is warranted to assess if this is the case. However, the findings suggest that few participants are confident in their knowledge of the system.

Few participants were aware of the structure of the kidney allocation system, believing that patients receive a kidney in a sequential order. In the absence of full knowledge of the allocation system, participants may attempt to make sense of the system by oversimplifying it through incorrectly anchoring it to their social representation of what constitutes a waiting list.^17^ Reference to being on a ‘waiting list’ may contribute to confusion about the way the system is structured. Perhaps replacing the term ‘waiting list’ with ‘waiting pool’ may therefore facilitate more appropriate understanding of how the allocation system works. Although the technical details of the allocation scheme are available online, few participants had accessed this information. Information is more effective if tailored to patients,^18^ and there is a risk that highly technical information may not be helpful to most patients. Health literacy is low in patients with kidney disease^19^ and patients' knowledge of their disease may be limited,^20^ so how best to make information available to patients may need to be reconsidered. In line with previous research from the USA,^21^ the majority of participants trusted that the allocation system was fair, while a minority of participants perceived a lack of transparency in how kidneys are allocated. Feeling ill-informed may lead to dissatisfaction with subsequent treatment. Despite this, the majority of participants said they were content with the information they received, so the provision of further medical detail may not be required. Instead, an explanation of how the factors inter-relate in the allocation process may be more useful. Managing expectations of transplantation affects how patients cope post-transplant,^22^ ^23^ so it is important that more information be provided about the weight given to matching, and the quality of the kidneys, to help manage patient expectations of transplant survival. Longer waiting times have been shown previously to be an important factor to UK patients,^8^ ^10^ but this was not found here. Participants believed that medical priority was the most important; where there is a good match it should be used regardless of waiting time, with a preference for waiting longer for a better-matched kidney. In a US study, Louis *et al*^16^ suggested that when patients have seen how detrimental a poor match can be, they may place more value on matching. In the current study, almost half of participants had at least one previous transplant, and all participants whose transplant failed considered matching to be of utmost importance. This may help explain the lack of weight given to waiting time by these participants.

Better matching and younger age were considered the most important factors in determining kidney allocation, which have been identified previously as important to Australian and UK patients.^7^ ^8^ Participants were more likely to prioritise children based on the expectation of increasing life expectancy, and being older was considered to bring more risk of having other conditions that may limit life expectancy, or even preclude transplantation. In contrast, a substantial minority of participants felt that no personal characteristics, including age, should be used to prioritise patients. The reason the current scheme prioritises younger patients for better-matched kidneys is in part to ensure that future transplantation is more easily achieved through avoiding development of antibodies to HLA. The provision of patient-friendly information would explain the reasoning behind prioritising particular groups, which may lead to greater acceptance and understanding of the factors used and of the system overall.

Participants felt that those who have the most medical need for a transplant should be prioritised. There was also acknowledgment that kidneys should be allocated to those who have the best chance of surviving and maintaining a functioning transplant.^7^ Allocating kidneys according to medical criteria was considered the best way to allocate organs, which has been found previously in liver transplantation where medical need is assumed to be an objective way to allocate organs, despite the fact that it may require moral judgement.^24^ Patients are aware of some of the issues surrounding allocation, but without complete information, patients cannot make well-informed judgements about policies. Previous scheme development has included patient representation, but providing further information to patients about the system may help them feel more confident of their knowledge and opinions as to how the system might be improved.

Although medical need and the use of medical criteria were seen to be important, non-medical and recipient factors were also discussed. For example, having children or dependants was one of the lowest-rated factors, but was raised repeatedly in the interviews. In line with our findings, there is inconsistency in the literature in preferences for this factor, with some UK studies indicating that patients wish to give greater priority to those with dependants,^9^ while an Australian study reported more mixed views.^7^ Those who do not believe that priority should be based on recipient factors were less likely to consider having dependants as worthy of prioritising patients for kidney allocation, so there is no consensus as to its perceived importance.

A small number of participants felt that patients whose kidneys were damaged by behaviours such as substance misuse were less deserving of a kidney transplant. Other research has shown that participants consider moral deservingness when discussing kidney transplantation.^7^ Previous research from the UK and the Netherlands has shown that kidney recipients reported feeling gratitude and a sense of duty to their donors to take care of their kidney,^22^ ^23^ so allocating transplants to those considered at risk of not taking care of their kidneys may explain why these factors were considered important in the current study.

The factors rated by participants at the end of the interview showed general agreement with the factors discussed in the interviews. Ratings varied widely, however. Participants found it difficult to distinguish the perceived importance of the factors listed with many showing ceiling effects. In contrast, participants were able to show distinct preferences when asked more general questions about how kidneys should be allocated in the interviews. Although participants were encouraged to elaborate on their ratings, few participants did so, although some of the factors had already been discussed in the interviews. Despite this, the differences in ratings and those factors discussed in interviews suggest that qualitative designs may be more effective in eliciting greater insight into participants' beliefs and preferences.

### Strengths and weaknesses of the study

The interviews were conducted with participants recruited to ATTOM, and as with all qualitative studies, the results cannot be generalised to all renal patients or other organ allocation schemes. No attempt was made to achieve a representative sample, so any frequencies given are not necessarily representative of the patient group as a whole*.* Moreover, comparisons between groups should be noted with caution, as the groups differed in demographic factors such as education level and ethnicity. The interviews were conducted with English-speaking participants from a subsample of transplant centres in the UK. The results therefore may not reflect fully the views of people of ethnic minority origin. Patients deemed unsuitable for transplant listing were not included in the present interview sample. Despite this, collecting qualitative data allowed us to delve further into patients' understanding of the allocation system. This is one of only a handful of studies examining patient beliefs about kidney allocation. Very little research has examined patients' views of kidney allocation and this study is timely to inform forthcoming guideline development and allocation scheme revision. Assessing a large group of patients who have yet to be listed would inform us of what patients know of the system before, during and after listing for a kidney transplant. This study does not provide a comprehensive understanding of patient knowledge, but instead provides a snapshot of such knowledge (or lack of knowledge) may relate to patient suggestions for the allocation scheme.

A representative survey to quantify knowledge, as well as issues regarding age priority, waiting time and the importance of dependants, would help further to describe and understand patient's beliefs and priorities. This study focused exclusively on renal patients; there is a case for considering the views of potential kidney donors and perhaps the wider population for their level of engagement and knowledge of transplantation issues.

## Conclusions

This study highlights that patients vary in their views and their priorities for kidney allocation. Most participants were uncertain of the accuracy of their knowledge. The majority of participants were aware that a number of factors are important, and most were content with their level of knowledge, so tailoring information to allow greater explanation of how factors in the allocation system relate to one another may be important in increasing patients' perceived ability to make informed decisions about the system. Although further work is warranted to assess it, replacing the term ‘waiting list’ with ‘waiting pool’ may perhaps facilitate more appropriate understanding of how a points-based allocation system works. Policymakers should continue to consult with patients, as it reveals patient knowledge and understanding, identifies information needs and provides guidelines for what factors may be considered acceptable to patients, which may help increase patients' confidence in being involved in treatment decision-making, and ultimately increase patient satisfaction.
