# Supplementary material for: Patient preferences, knowledge and beliefs about kidney allocation: qualitative findings from the UK-wide ATTOM programme
Source: BMJ Open. 2017 Jan 27;7(1):e013896. doi: 10.1136/bmjopen-2016-013896 (PMC5278279; doi:10.1136/bmjopen-2016-013896)

Supplementary files

Appendix 1. Flow diagram of participant recruitment and selection.

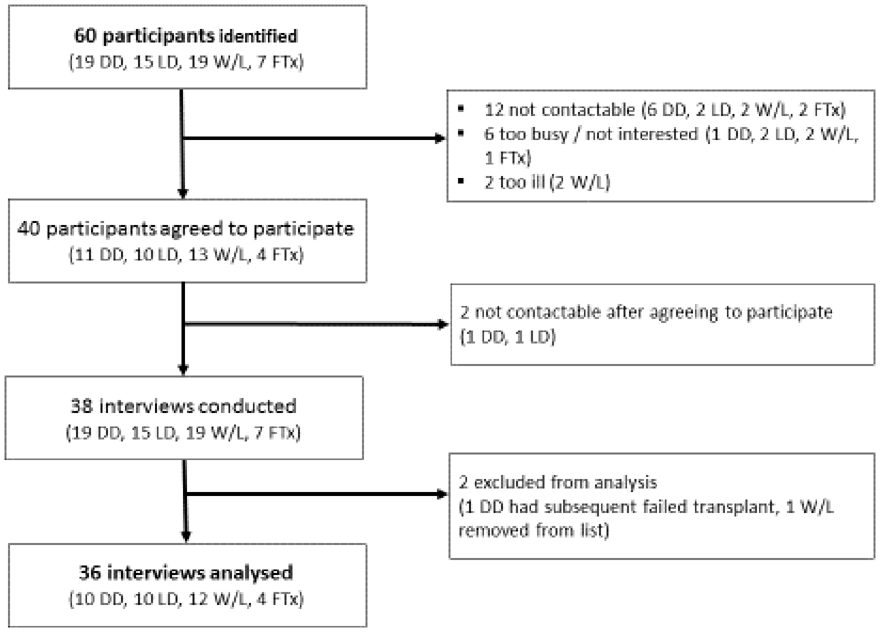

Appendix 2. Diagram of themes

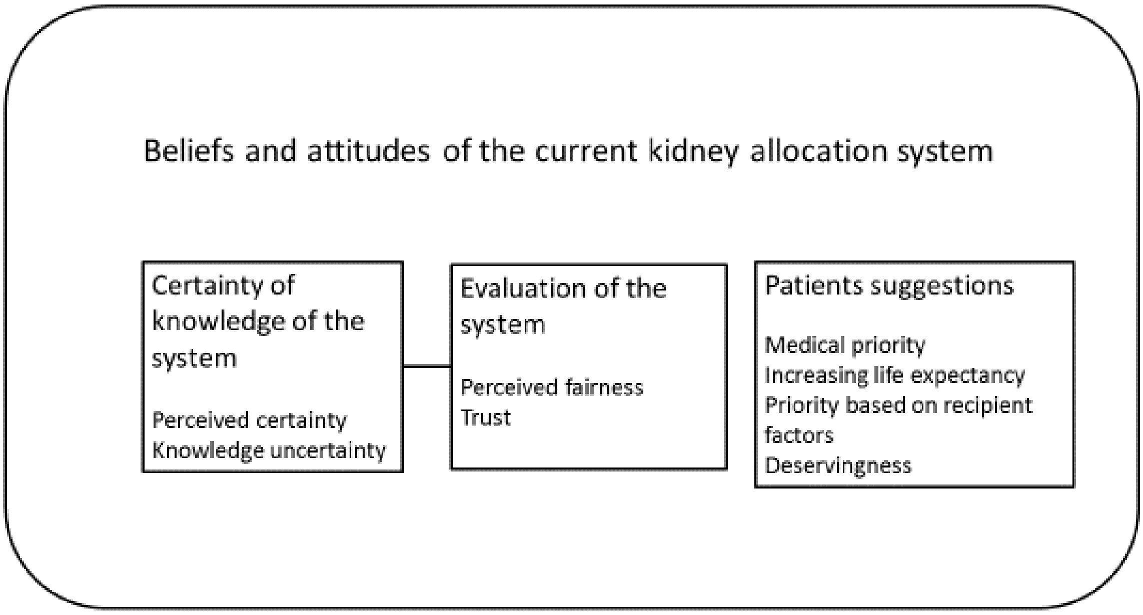

Supplement: supplementary appendices [file bmjopen-2016-013896supp_appendices.pdf]
